# RhoB: Team Oncogene or Team Tumor Suppressor?

**DOI:** 10.3390/genes9020067

**Published:** 2018-01-30

**Authors:** Julia A. Ju, Daniele M. Gilkes

**Affiliations:** 1 Department of Oncology, The Sidney Kimmel Comprehensive Cancer Center, The Johns Hopkins University School of Medicine, Baltimore, MD 21231, USA; jju4@jhu.edu; 2 Department of Chemical and Biomolecular Engineering, The Johns Hopkins University, Baltimore, MD 21218, USA

**Keywords:** cancer progression, tumor suppressor genes, oncojanus genes, oncogene, RhoB, Rho GTPases

## Abstract

Although Rho GTPases RhoA, RhoB, and RhoC share more than 85% amino acid sequence identity, they play very distinct roles in tumor progression. RhoA and RhoC have been suggested in many studies to contribute positively to tumor development, but the role of RhoB in cancer remains elusive. RhoB contains a unique C-terminal region that undergoes specific post-translational modifications affecting its localization and function. In contrast to RhoA and RhoC, RhoB not only localizes at the plasma membrane, but also on endosomes, multivesicular bodies and has even been identified in the nucleus. These unique features are what contribute to the diversity and potentially opposing functions of RhoB in the tumor microenvironment. Here, we discuss the dualistic role that RhoB plays as both an oncogene and tumor suppressor in the context of cancer development and progression.

## 1. Introduction

Rho proteins are small molecules (~21 kDa) that belong to the Ras superfamily and function as binary switches in a wide variety of signaling pathways [1]. They consist of a family of 20 intracellular signaling molecules including RhoA, RhoB, RhoC, RhoG, RhoE, Rac1, Rac2, Cdc42Hs, and TC10 that are most well-known for their role in regulating the actin cytoskeleton [2]. However, they are also known to activate pathways that regulate gene transcription, vesicle trafficking and cytoskeletal reorganization, which are all processes that control growth, differentiation, adhesion, and migration of cells. To stimulate these pathways, the small GTPases must be in their active, GTP-bound conformation. Only in this state are they able to bind effector proteins and transduce signals from membrane receptors such as cytokine and growth factor receptors, integrins and G-protein coupled receptors [3]. 

**Figure 1:**
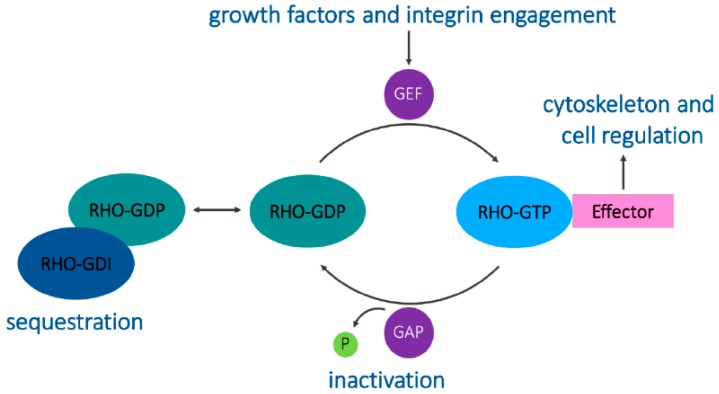
The Rho GTPase cycle. Guanine nucleotide exchange factors (GEFs) catalyze the exchange of GDP for GTP to activate Rho GTPases. GTPase-activating proteins (GAPs) promote GTP hydrolysis to decrease Rho GTPase activity. Rho GDP-dissociation inhibitors (GDIs) sequester Rho GTPases in the cytoplasm, maintaining them in an inactive state. Active Rho GTPases act on their downstream effectors to engage specific signaling cascades.

A structural feature that distinguishes the Rho proteins from other small GTPases is the Rho insert domain located between a β strand and an α helix within the small GTPase domain [1]. Typically, Rho proteins are 190–250 residues long and consist of only the GTPase domain and short terminal C-terminal extensions. They all contain the sequence motifs characteristic of all GTP-binding proteins and cycle between binding to GDP and GTP. This cycle is regulated by guanine nucleotide exchange factors (GEFs) that allow GTP binding by displacing the bound GDP in the active site and by GTPase-activating proteins (GAPs) that allow the hydrolysis of bound GTP, switching the GTPase to an inactive conformation [1,4]. These GEFs and GAPs often facilitate the integration of Rho GTPases and other signaling proteins together with their downstream targets [2]. Guanine nucleotide-dissociation inhibitors (GDIs) bind to C-terminal prenyl groups on Rho proteins, sequestering them in the cytoplasm away from their regulators and targets (Figure 1) [5]. 

Additionally, most of the Rho family members undergo C-terminal post-translational modifications by isoprenoid lipids, which in turn regulate their subcellular location and function [1]. Rho GTPases have been implicated in many cellular processes including actin and microtubule cytoskeleton organization, cell division, motility, cell adhesion, vesicular trafficking, phagocytosis and transcriptional regulation [2].

The Ras superfamily of proteins are mutated in about 30% of human cancers, and recent genetic studies by whole genome sequencing have identified an expanding list of recurring mutations in Rho GTPases (reviewed extensively in [6]), which was previously thought to be rare in cancers. Deregulation of Rho GTPase signaling can also occur at the level of expression or activation where the level of Rho GTPase expression or activation is altered by upstream regulators or downstream effectors [3]. In vitro studies have shown that Rho-GEFs are more potent oncogenes than GTPase-defective Rho proteins, and that a fast GTP-GDP cycling mutant of Cdc42 (a Rho family member) has a greater transforming capacity than a GTPase-defective mutant [7]. A fast GTP-GDP cycling mutant contains significantly increased intrinsic GDP/GTP activity and remains responsive to Rho-GAP stimulation to cycle between the GDP and GTP-bound states, whereas the GTPase-defective mutants cannot hydrolyze bound GTP and are locked into the GTP-bound active conformation and are therefore constitutively active. Both types of mutants result in the net enhancement of active Rho-GTP species in cells and have been widely used in studying the activity of small GTPases. Both mutant forms of RhoB that were introduced into NIH3T3 cells were effective in stimulating actin stress fiber formation and focal complex assembly and showed a potent transforming activity in a foci-forming assay [8]. The correlation between Rho-protein expression and clinical outcome suggested the potential use of their expression levels as prognostic indicators [9].

RhoA, RhoB and RhoC proteins share about 85% amino acid identity, yet they each play unique biological roles [10]. Differences in their subcellular localization most likely lead to separate effector interactions and therefore a diverse set of functions. It has been well-established in many studies that RhoA and RhoC are pro-tumorigenic in almost all cancer types [11,12,13,14]. RhoB, however, is the least studied amongst its immediate family members, especially in the context of tumorigenesis and cancer progression [15]. Because of the several unique post-translational modifications that are distinct from RhoA and RhoC, RhoB is argued to be the most diverse protein of the Rho subgroup, a factor possibly contributing to its dualistic role in cancer [16].

## 2. Structure and Localization of RhoB

**Figure 2:**
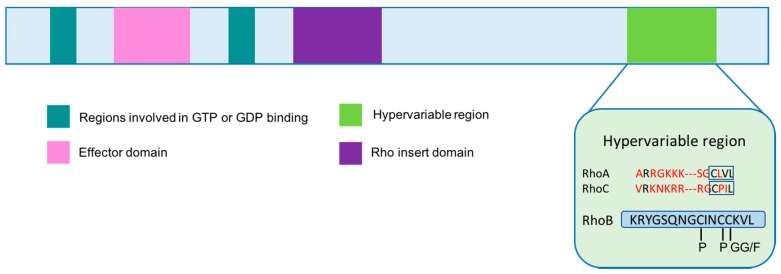
Rho GTPase domain organization. The differences between RhoA, RhoB and RhoC lie in the hypervariable region. Red amino acid residues indicate differences from RhoB sequence. RhoB can be palmitoylated, farnesylated and geranylgeranylated, where RhoA and RhoC can only be palmitoylated. P: palmitoylation; GG: geranylgeranylation; F: farnesylation. Adapted from [16].

RhoB has several additional features that are distinct among Rho proteins. First, the *RhoB* gene is smaller and contains only one exon, which initiated the hypothesis that RhoB may have evolved by reverse transcription [17]. RhoB also contains a unique C-terminal region that undergoes specific post-translational modifications affecting its localization and function [8]. While RhoA and RhoC can only be palmitoylated, RhoB can also undergo farnesylation (RhoB-F) or geranylgeranylation (RhoB-GG) and its prenylation state affects its function [11] (Figure 2). In contrast to RhoA and RhoC, RhoB not only localizes at the plasma membrane, but also on endosomes, multivesicular bodies and has even been identified in the nucleus [2]. Farnesylated RhoB tends to localize to the cell membrane, promotes cell growth, mediates the effects of Ras on actin cytoskeleton, and activates nuclear factor kappa B [18,19,20]. In contrast, geranylgeranylated RhoB localizes to endosomes and induces cell apoptosis [18,21]. This distinction in subcellular compartmentalization is likely due to its differential prenylation. All of these characteristics ultimately contribute to RhoB’s role in the regulation of proliferation, survival, invasion and angiogenic capacity.

## 3. RhoB Regulation and Expression

RhoB, similar to the other Rho GTPases, functions as a molecular switch that cycles between an inactive GDP-bound form and an active GTP-bound form [6]. *RhoB* transcript has a half-life of only 30 min and its accumulation fluctuates significantly throughout the cell cycle. A mechanism to stabilize *RhoB* mRNA is mediated by its interaction with the RNA-binding protein HuR [22]. RhoB protein turnover rate is high and has a half-life of only 2 h. RhoB expression is rapidly induced by various stimuli including UV radiation, cytokines, growth factors, genotoxic stress, steroid and toxin treatments suggesting it may be highly responsive to stress-induced signaling events [23,24,25]. RhoB is known to be part of the immediate early genetic response to epidermal growth factor, transforming growth factor β, Src activation, or genotoxic stress [26,27,28,29]. RhoB was shown to have potential implications for EGF signaling by targeting the activated EGF receptors to the lysosome, which represents an “off-switch” for mitogenic signals [29]. RhoB was also demonstrated to exert a negative regulatory influence on TGF-β-induced transcriptional activation [26]. The activity of the *RhoB* promoter was stimulated by genotoxic treatments indicating its role in the cellular response to DNA damage [27,28]. In addition to this, some studies show that Ras actually downregulates RhoB via the EGFR, ErbB2, and AKT/PKB pathways [30]. Epigenetic changes have also been proposed to regulate the *RhoB* promoter such as histone deacetylase-1 (HDAC1) repressing RhoB expression [31]. Inversely, it was shown that cells treated with farnesyltransferase and geranylgeranyl transferase inhibitors (FTIs and GGTIs) induced the transcription of *RhoB* by inducing HDAC1 dissociation and promoting histone acetylation of the *RhoB* promoter mediated by p300, a histone acetyltransferase [32]. It was reported that NF-Y, c-Jun and p300 are recruited to the *RhoB* promoter in response to UV irradiation or FTIs resulting in the transcriptional regulation of RhoB [33,34]. It was also hypothesized that direct cross-talk between HDAC6 and p300 could be a mechanism to regulate *RhoB* gene transcription upon HDAC6 inhibition [35]. Further, HDAC inhibitors that are known to kill tumor cells, were also shown to induce *RhoB* expression [36,37,38]. This suggests that the re-expression of *RhoB* alone by inhibiting HDAC expression is not sufficient to promote tumor progression. 

## 4. Signaling and Trafficking by RhoB

The family of Rho GTPases was originally characterized as regulators of the actin cytoskeleton, and their diverse functions stem from a foundation in actin organization. Rho proteins influence trafficking of endosome-receptor signaling complexes, which leads to either lysosomal degradation or recycling to the plasma membrane and even the nucleus [39]. RhoB was the first member of the Rho family to be implicated in endosomal trafficking. One study showed that RhoB localizes and activates its downstream target, serine/threonine kinase (PRK1), on endosomes [40], and acts through this signaling pathway to disrupt the trafficking of internalized EGF receptor from endosomes to a prelysosomal compartment. RhoB was shown to be vital for plasma membrane recruitment and activation of the Src tyrosine kinase [41] and essential to maintain a nuclear trafficking pathway for Src that was stimulated by PDGF in vascular smooth muscle cells [42]. Finally, RhoB was also shown to affect nuclear trafficking of AKT/PKB as well as the phosphorylation of AKT in endothelial cells, potentially regulating endothelial cell survival [43]. The substantial roles that RhoB seems to play in vascular and endothelial cells presumably gives rise to involvement in cancer vasculature with implications in angiogenesis.

## 5. RhoB in Cancer

RhoB was first described as contributing to Ras-induced fibroblast transformation. Using a dominant negative *RhoB* gene that was mutated on the amino acid residue 19 from an asparagine to a threonine, Prendergast et al. showed that oncogenic Ras-induced cell focus formation required RhoB [19]. The group went on to demonstrate that NIH3T3 cells, transfected with a vector encoding an activated *RhoB* gene, grew to a higher saturation density and displayed reduced serum and anchorage requirements for growth. Soon after, the Prendergast group demonstrated that the overexpression of a geranylgeranylated form of RhoB, was required for the FTI-mediated apoptotic response of Ras-transformed cancer cells [44,45]. Subsequent studies began to reveal emerging evidence for a cancer suppressive role for RhoB through inhibitory effects on cell proliferation, survival, invasion and metastasis [46,47]. These in vitro observations were supported by in vivo findings that RhoB depleted cells formed tumors more efficiently than cells expressing RhoB when injected intraperitoneally into mice [48]. Additionally, cells transfected with different RhoB constructs (only RhoB-F, only RhoB-GG or both RhoB-F/GG) and subcutaneously implanted into the flank of nude mice suppressed tumor growth when compared to the corresponding empty vector [15]. The difference in these findings has led to controversy as to whether RhoB promotes or suppresses tumor growth. Table 1 highlights this contrasting role of RhoB in several cancer types. Whereas RhoA and RhoC proteins have been shown to have a positive role in proliferation and malignant transformation, the specific role of RhoB in these processes appears to be much more complex [49]. Because of this, it is highly likely that RhoB functions in a contextual manner, responding to specific signals in the tumor microenvironment. Since the tumor microenvironment is composed of several different cell types, including endothelial cells and fibroblasts, the way these cells respond to changes in RhoB may differ. To investigate *rhoB* deletion on tumor formation, mouse embryonic fibroblast cells transformed by adenovirus E1A plus mutant RAS with a single copy of *rhoB* deleted (ER^+/−^) or a homozygous deletion of *rhoB* (ER^−/−^) were injected into the intraperitoneal cavity of syngeneic Sv129 mice [48]. The results reported from this experiment show an increase in tumor nodule formation from ER^−/−^ injected cells when compared to ER^+/−^ cells. However, an alternative mouse model utilizing a xenograft assay of ER^+/−^ or ER^−/−^ cells subcutaneously injected into opposite thighs of immunocompromised *scid* mice showed no difference in tumor formation [21]. This further highlights that the function of RhoB may depend on the influence of the microenvironment, such as immune cells, and how they interact with the tumor cells. Alternatively, it highlights the possibility that different experimental growth assays (growth on the thigh of a mouse versus into the intraperitoneal cavity) may yield different results. 

## 6. Oncogenic Role of RhoB

Studies have shown that RhoB contributes to tumor formation by stimulating proliferation, angiogenesis, invasion and migration. The following is a review of several studies which highlight the role of RhoB as an oncogene in different cancer types. 

### 6.1. Glioblastoma

Gliomas are the most common malignant primary brain tumor in adults and patients with glioblastoma have a median survival time of 14 months [50]. One reason for this poor survival time is due to glioma’s highly invasive nature, which prevents complete surgical removal of all malignant cells and imposes great limitations to localized radiation therapy [51]. RhoB depletion leads to cell cycle arrest, apoptosis and reduced tumorigenic potential of glioblastoma cells in vivo [52]. Ma et al. show that RhoB supports tumor tolerance to cellular and environmental stresses, in part via the p53 and STAT3 pathways [52]. However, RhoB overexpression did not promote glioblastoma cell growth or affect p53 or STAT, suggesting that RhoB is not a rate-limiting oncogenic factor in glioblastoma, unlike a typical oncogene. A recent study implicated RhoB as a possible regulator in a signaling pathway that would enhance the ability for glioma cells to adapt to hypoxia [53]. It is well-known that tumor hypoxia, and subsequently an increase in HIF-1α expression, results in a poor patient prognosis [54,55,56]. Using small interfering RNAs (siRNAs) against RhoB, Skuli et al. showed that silencing RhoB under hypoxic conditions inhibited HIF-1α accumulation in a proteasome-dependent manner as well as inhibited the transcriptional activity of HIF-1α as evidenced by a decrease in CA-9 expression [53]. Reducing RhoB expression under hypoxia resulted in a decrease in phosphorylated AKT, a decrease in the phosphorylated Ser^9^ inactive form of GSK-β and an increase in its phosphorylated Tyr^216^ active form. To understand the upstream mechanisms of RhoB, they looked at how hypoxia influenced RhoB expression or activity and found that hypoxia did not change the level of RhoB in cells, but did increase the amount of activated RhoB. Using a NADPH oxidase inhibitor, they concluded that hypoxia activates RhoB via ROS production. These results suggest that RhoB is activated in response to a decrease in intracellular oxygen levels, which in turn activates hypoxia-inducible pathways. The authors previously showed that inhibiting RhoB, using the dominant-negative mutant RhoBN19, in U87 human glioblastoma xenografts increased their radiosensitivity, partially by increasing the oxygenation in the tumors [57]. These studies also highlight that the pro-tumorigenic/anti-tumorigenic effects of RhoB may be context-dependent. For example, the role of RhoB may be different in hypoxic versus non-hypoxic tumor environments. However, caution should be taken to extrapolate these results to other tumor types due to the context-dependency of RhoB function as well as potential differences in the response to hypoxia.

### 6.2. Breast Cancer

Rho protein overexpression is known to occur in breast tumor tissue and has been correlated with poor clinical outcomes in breast cancer [58]. Fritz et al. demonstrated that enhanced protein levels of RhoB were found in breast tumors when compared to the corresponding normal tissue [59]. This overexpression with respect to normal tissue correlates with tumor grade where high RhoB expression was found in grade III ductal breast cancers as compared to grade I. Consistent with these findings, Médale-Giamarchi et al. showed a positive crosstalk between RhoB expression and ER-α expression and the significant role of RhoB in regulating the proliferation of ER-α positive breast cancer cells [60]. They found that RhoB expression was highly correlated with ER-α and PR expression and that RhoB modulates ER-α expression by controlling both its protein and mRNA levels. Their results provide evidence for pro-oncogenic function in hormone-dependent breast cancer cells. 

A study done by Kazerounian et al. demonstrated that RhoB differentially controls AKT function in tumor versus endothelial cells, resulting in decreased proliferation of early stage cancer that was superseded by the proangiogenic functions of RhoB in endothelial cells during tumor progression [61]. Using the MMTV-PyT mouse model interbred with *rhoB* null mice, the authors found an increase in the number of early tumor lesions in *rhoB*^−/−^ animals, where *rhoB* is deleted in both the tumor and endothelial cells. Three-dimensional growth of isolated *rhoB*^−/−^ tumor cells revealed enhanced growth in acini compared to *rhoB*^+/−^ cells and increased cell proliferation compared to *rhoB^+/+^* cells. Similarly, shRNA-mediated reduction of RhoB in MDA-MB-231 breast cancer cells displayed a hyperproliferative phenotype, which was actually associated with decreased invasion into a matrigel matrix. In a previous study, the authors showed a positive regulation between RhoB and AKT activity in nontumor endothelial cells [43]. In contrast, this study revealed in both tumor tissues, tumor cells isolated from *rhoB*^−/−^ mice and shRhoB MDA-MB-231 cells, increased levels of activated p-AKT were detected compared with the respective controls, potentially promoting a tumor suppressing function of RhoB. However, when assessing tumor progression, the greater number of early lesions initially observed in the *rhoB*-deficient mice did not result in greater overall tumor burden at the endpoint of the experiment. Instead, *rhoB*-expressing animals had larger, faster growing tumors compared to the *rhoB*-deficient transgenic mice. The opposite roles of RhoB in p-AKT regulation suggest contrasting biological function between tumor cells and nontumor endothelial cells. Tumors stained with CD31, an endothelial cell marker, illustrated a significant decrease in the tumor vasculature of *rhoB*^−/−^ mice. Isolating endothelial cells from tumors in the *rhoB*^−/−^ and *rhoB^+/+^* mice, showed a dose-dependent decrease in p-AKT associated with allelic loss, supporting the idea that RhoB may play competing biological roles in tumor versus endothelial cells. Wild-type (*rhoB^+/+^*) tumors grown in *rhoB*^+/−^ and *rhoB*^−/−^ hosts demonstrated that the RhoB-deficient stroma was adequate to restrict tumor growth of *rhoB^+/+^* cells. *rhoB*^−/−^ endothelial cells also had lower VEGF-A expression and demonstrated impaired angiogenesis ability. Then, a reciprocal experiment where *rhoB*^+/−^ and *rhoB*^−/−^ tumors were grown in wild-type hosts demonstrated that *rhoB* loss in tumor cells was sufficient to reduce tumor growth even in a *rhoB^+/+^* environment. Moreover, this shows that within the same tumor RhoB can have both a tumorigenic and tumor suppressive role depending on the cell type and that it is possible for one function to dominant over the other. It also underscores the complicated challenge in developing therapeutics to target RhoB, since most therapies tend to target both the tumor and stroma in the same way.

### 6.3. Lung Adenocarcinoma

In lung adenocarcinoma, Luis-Ravelo et al. identified *RhoB* as a gene that promotes early and late stages of metastasis [62]. In this study, using intracardiac injection of A549 cells expressing a RhoB knockdown construct, the authors demonstrated a significant decrease in metastatic bone colonies with smaller bone lesions. By intratibial injection of shRhoB and control cells, the authors further demonstrated that shRhoB cells had an impaired ability to colonize and adapt in the bone compartment, which suggests a possible mechanism for the decreased metastatic activity of shRhoB cells. RhoB-overexpression did not alter the growth of orthotopic tumors, but did result in the detection of some extra pulmonary tumors. Consistent with the in vivo findings, RhoB-depleted cells exhibited a decreased invasion capability in collagen and was associated with an increased sensitivity to paclitaxol. On the other hand, cells with overexpressed RhoB levels showed increased resistance to paclitaxel both in vitro and in vivo. Based on this finding, RhoB could be considered to contribute to an aggressive metastatic phenotype as well as resistance to therapy.

Although several EGFR-tyrosine kinase inhibitors (TKIs), such as erlotinib, gefitinib and afatinib, have been clinically approved to treat metastatic lung cancer, only seventy percent of patients that harbor EGFR-mutated tumors respond to these inhibitors. Almost all patients eventually develop irreversible resistance. In a recent study by Calvayrac et al., low levels of RhoB expression in the primary tumor of patients was correlated with a good response to EGFR-TKI treatment, while high levels of RhoB corresponded to a poor response [63]. They also found that a gradual increase in RhoB expression, through recombinant adenoviral transfection, gradually increased the erlotinib IC_50_ values in an EGFR-mutated lung cancer cell line. In mouse models, inhibiting AKT with G594, a selective AKT inhibitor, reversed RhoB-induced resistance to erlotinib. Further experiments demonstrated that combining G594 with erlotinib could sustain the sensitivity of cells overexpressing RhoB to the EGFR-TKI. The results of this investigation suggest that the levels of RhoB in lung tumors can potentially predict resistance to EGFR-TKIs in patients that have an EGFR-mutation and offer a new strategy to increase the patient response rate with a combination of inhibitors. 

### 6.4. Other Cancers

Emerging evidence continues to suggest potential roles of RhoB in supporting tumorigenic functions. For example, RhoB expression is upregulated in T-acute lymphoblastic leukemia (T-All) cells in comparison with normal T cells and is significantly correlated to white blood cell counts [64]. Overexpression of RhoB enhanced cellular movement and invasion of prostate cancer cells in vitro, mediated by GSK-3 signaling and MMP1-dependent invasive potency in collagen gels [65]. The authors speculate that the mechanism between RhoB and MMP1 expression might involve MUC1 phosphorylation by GSK-3β. Additionally, knockdown of RhoB has been described to induce an apoptotic response in renal cells [66]. In this study, inhibiting RhoB led to a greater than 300% increase in cell apoptosis and a relocalization of focal adhesion kinase. In hepatocellular carcinoma (HCC) cells, CCL24, a chemotactic factor, was shown to promote migration and invasion via a RhoB-VEGFA signaling pathway [67]. Activation of RhoB appears to protect cells from radiation-induced apoptosis in HeLa cells by preventing post-mitotic cell death [68]. RhoB also protected keratinocytes from UVB-induced apoptosis via EGFR signaling [69]. Delmas et al. demonstrated that in BRAF-mutant melanoma cells, inhibition of BRAF or its target, MEK, induces RhoB expression by a c-Jun dependent mechanism [70]. The authors also demonstrated that RhoB deficiency resulted in hypersensitivity to BRAF and MEK inhibitors. Further, they revealed that loss of RhoB expression in metastatic melanoma tissues is correlated with an increase in progression-free survival of BRAF-mutant patients that were treated with vemurafenib. Mechanistic studies revealed that RhoB modulates the response of melanoma cells in vitro to PLX4032, a different BRAF kinase inhibitor, via the AKT pathway and combination of an AKT inhibitor with PLX4032 in vivo reversed RhoB-mediated resistance to PLX4032. The study concluded that PLX4032-induced RhoB expression drives AKT-dependent cell survival and using combination therapies could potentially improve response rate of tumors with positive RhoB expression.

## 7. Tumor Suppressive Role

Although early studies indicated that RhoB had a positive role in cell growth, more recent investigations have shown that RhoB is downregulated in some tumors, suggesting that RhoB may also act as a tumor suppressor.

### 7.1. Lung Cancer

Loss of RhoB expression also occurs very frequently in lung carcinogenesis as shown by many studies [37,49], and has been correlated with poorer outcomes [37], reinforcing its potential role as a tumor suppressor. By analyzing RhoB expression in human lung tissue ranging from normal to invasive carcinoma, Mazieres et al. demonstrated in two independent cohorts of patients that RhoB expression decreased with lung cancer progression as well as inversely correlated with the proliferation marker, Ki-67 [49]. Using lung cancer cells transfected with either an empty vector or a RhoB-overexpressing vector subcutaneously injected into nude mice, the authors confirmed this observation by showing a dramatic delay in tumor growth in the mice bearing tumors that overexpressed RhoB. In another study, the *RhoB* gene was found to be located in the homozygous deletion region of chromosome 2p24 that shows frequent allelic loss and downregulation in NSCLCs [37]. By treating NSCLC cell lines with low *RhoB* expression with the HDAC inhibitor, trichostatin (TSA), they showed a substantial gene reactivation in the cell lines, suggesting the epigenetic regulation of *RhoB*. As previously demonstrated [61], the context of RhoB expression may explain the apparent contradicting observations between these studies and the ones mentioned above supporting RhoB function as a tumor promoter. 

### 7.2. Skin Cancer

RhoB expression was reduced in melanoma cells when compared to primary human melanocytes [71]. Jiang et al. revealed that RhoB is suppressed by AKT in melanoma cells and that downregulation of RhoB is a crucial step in oncogenic transformation [30]. Treatment of B16-F10 melanoma cells with LY294002, a PI3K inhibitor, strikingly induced RhoB, but not RhoA protein levels. Intravenous injection of B16-F10 melanoma cells overexpressing RhoB, but not RhoA, inhibited colonization of melanoma cells in the lung in their mouse model. Their results support an antagonistic interaction between the oncogenic Ras/PI3K/AKT pathway and RhoB. Although RhoB was also shown to be dispensable for mouse development, *rhoB* nullizygous mice exhibited accelerated chemically induced skin tumors and increased efficacy of intraperitoneal tumor formation [48]. In a more recent study, *rhoB* deletion (*rhoB*^−/−^) lowered the risk of UVB-induced skin carcinogenesis in mice, but the tumors that did form were undifferentiated and more proliferative, indicating that RhoB may promote the initiation of skin cancer while at the same time limiting the tumor aggressiveness [72]. Further, human SCC tumor samples stained for phosphorylated histone H2AX, a marker for DNA double stranded breaks (DSBs), revealed the undifferentiated and RhoB-deficient human skin tumors had elevated levels of γH2AX expression. These data indicate that a loss of RhoB corresponds to increased levels of DSBs, which suggest that reduced expression of RhoB could contribute to genetic instability and therefore contributing to skin tumor progression. 

### 7.3. Brain Cancer

In contrast to observations described above that revealed an oncogenic role for RhoB in glioblastoma, Forget et al. reported that RhoB protein expression levels are inversely related to tumor malignancy in a series of 24 human brain tumors including astrocytomas, anaplastic astrocytomas, glioblastomas and pilocytic astrocytomas [73]. Additionally, they suggest that based on their results, RhoB could be defined as a molecular marker to differentiate particular types of brain cancer according to their histological grade. Current histological diagnoses of brain tumors are complicated by similarities between the different grades, therefore, the idea of having a molecular confirmation with RhoA and RhoB could greatly improve the therapeutic approach. To support this, a later study done by Baldwin et al. showed PKCι negatively regulates the expression of RhoB, and that restoration of RhoB expression inhibited glioblastoma cell motility and invasion [74]. Inhibition of PI3K with LY294002 resulted in a decrease in PKCι phosphorylation and an increase in RhoB expression, while inhibition of ERK had no effects on RhoB levels. Conversely, they also demonstrate that RhoB constitutive expression can suppress PKCι activity, implying the two proteins could be mutually antagonistic and function as a switch for glioblastoma cell motility. Their results suggest that abnormal activation of the PI3K pathway in glioblastoma represses RhoB expression via PKCι activation, indicating PKCι as a potential drug target for glioblastoma therapy.

### 7.4. Ovarian Cancer

RhoB expression is decreased or lost in ovarian carcinoma and decreases significantly with increasing grade [36]. This study revealed that HDAC inhibitor, trichostatin (TSA), significantly increased RhoB expression, resulting in decreased proliferation and inhibition of cell cycle progression of ovarian cancer cells whereas the methyltransferase inhibitor, 5-Azactidine, had no effects on these functional properties. Because of this, the authors propose that *RhoB* expression is most likely regulated by histone deacetylation rather than promoter hypermethylation. Vishnu et al. showed that sunitinib, a RTK inhibitor, leads to upregulated *RhoB* mRNA expression by an unknown mechanism and that the synergistic effects of sunitinib and ixabepilone on apoptosis are abrogated by silencing RhoB in both naïve and paclitaxel-resistant cells [75]. Based on these results, they indicate that RhoB could be used as a potential early biomarker of response to similar combination therapy. Additionally, RhoB overexpression in vitro, through recombinant adenovirus transduction, resulted in a decrease in ovarian cell proliferation and induced apoptotic cell death within 24 h post-transduction, as shown by an increase in Apo2.7 positive cells and an increase in cleaved caspase-3 [76]. Further, in vivo restoration of RhoB led to the suppression of tumorigenicity, providing evidence in favor of reactivation of the RhoB signaling pathway to enhance ovarian cancer therapy.

### 7.5. Other Cancers

Studies of patient biopsies revealed that RhoB expression levels are also dramatically decreased in bladder cancer [77], cervical cancer [78], colorectal cancer [79], gastric cancer [80], head and neck cancer [81], kidney cancer [82] and pancreatic cancer [83]. In carcinomas in situ and in well-differentiated head and neck tumors, RhoB expression is quite prevalent [81]. However, this expression becomes weak and even undetectable as the tumors become highly invasive and more undifferentiated. Zhou et al. demonstrated that RhoB was significantly reduced or absent in gastric cancer tissues and significantly inhibited proliferation, migration and invasion of gastric cancer cells [80]. Additionally, they determined that RhoB expression induced apoptosis and enhanced chemosensitivity of these cells to anticancer drugs. In a recent study, Chen et al. established that RhoB was significantly downregulated in ccRCC tissues and cancer cell lines and that RhoB expression was inversely correlated with tumor size [82]. Their results showed that a decrease in RhoB expression triggered a higher malignancy potential in ccRCC with important roles in proliferation, cell cycle progression, apoptosis and migration. This suggests that RhoB may be an indicator of ccRCC progression and using RhoB as a biomarker could improve the efficiency of current therapeutic strategies. Additionally, ligand-bound thyroid hormone receptor beta (TRβ) was found to activate the RhoB signaling pathway upstream of p21 in thyroid cancer cells resulting in an inhibition of proliferation both in vitro and in vivo that required upregulation of RhoB [84].

## 8. Angiogenic Role

When studying the roles of potential oncogenes or tumor suppressor genes, it is important to consider not only the expression changes that occur in cancer cells themselves, but also the non-transformed cells in the tumor microenvironment. These surrounding cells such as immune cells, fibroblasts, inflammatory cells, lymphocytes and endothelial cells undoubtedly play a role in malignant transformation. Emerging studies have suggested a pivotal role for RhoB in the regulation of vascular function and angiogenesis. It has been well-documented that angiogenesis is a major contributing factor to cancer progression, so it is not surprising that several studies look at endothelial response to changes in expression or activation of RhoB. Using siRNAs, RhoB has been shown to be required for endothelial cell migration, sprouting and capillary morphogenesis [89]. *rhoB* knockout in mice leads to apoptosis of newly formed retinal vasculature via AKT nuclear exclusion and degradation [43]. Likewise, depletion of RhoB activity and expression in neonatal rats and primary endothelial cell culture is associated with apoptosis in the sprouting ECs of newly forming vessels. Another study revealed that RhoB silencing impaired endothelial cell migration and tubulogenesis via miR-21 targeting of *RhoB*, providing a potential mechanism for miR-21 to inhibit angiogenesis [90]. Additionally, RhoB has been shown to negatively regulate endothelial barrier reformation, critical in the inflammatory response, through the control of Rac1 localization and activity [91]. Impairment of barrier reformation, a critical underlying problem in chronic inflammatory diseases, was due to inhibition of Rac1 trafficking to the cell border by RhoB, whereas induction of Rac1 translocation to the plasma membrane accelerated recovery. HUVECs cultured with conditioned media from Huh7-CCL24-siRhoB HCC cells, having reduced RhoB expression, showed a significant decrease in tube formation ability versus the Huh7-CCL24 cells with normal RhoB expression in a VEGFA dependent manner [67]. In resting endothelial cells, reduction of RhoB via siRNA increased VE-cadherin levels at cell-cell contacts to promote enhanced barrier function under both normal and thrombin-induced conditions [92]. Utilizing a double knockdown, the authors were able to show that the reduced expression of both RhoA and RhoB combined, protected against thrombin-induced permeability. These findings indicate that RhoB is an essential regulator of endothelial barrier function under basal conditions.

The study conducted by Kazerounian et al. that is mentioned above in more detail, explores this role of RhoB in both the tumor and the surrounding endothelial cells. This study highlights how in tumor cells, RhoB functions as a tumor suppressor that inhibits Akt signaling, whereas in endothelial cells, RhoB harbors a tumor promoter role by sustaining endothelial AKT signaling [61]. This investigation underscores a dual role for RhoB in tumor progression. The mechanisms behind how RhoB affects endothelial cells are not completely understood. One possibility is that RhoB acts through the actin cytoskeleton where it is a main regulator of stress fiber formation [90,93]. Another possibility is that RhoB could regulate the activity of other GTPases such as RhoA or by regulating growth factor receptor trafficking and signaling [89]. Taken together, these observations suggest exploring the function of RhoB in tumor vascularity is necessary to provide answers on its affect throughout the overall process of tumor initiation and metastasis.

## 9. RhoB in FTI Anticancer Response

RhoB was not highly investigated until several studies indicated that it may play a role in the response to farnesyltransferase inhibitors (FTIs). FTIs were initially developed as a therapy to inhibit Ras function in tumor cells by blocking its post-translational farnesylation [10]. Ras was one of the first reported oncogenes and its role in inducing malignant transformation is very well-documented. [94]. The Ras oncogene was also reported to confer resistance to ionizing radiation [95,96]. However, mechanistic studies soon revealed that the cellular response to FTIs extended beyond its roles in targeting Ras function [97,98]. Subsequent studies showed that treatment of tumor cells with a FTI leads to a decrease in farnesylated RhoB levels and a corresponding increase in RhoB-GG levels [44]. This increase in RhoB-GG was shown to be sufficient and sometimes necessary for FTIs to initiate phenotypic alterations, cytoskeletal actin reorganization, growth inhibition, and apoptosis [21,44,45]. Whereas farnesylated RhoB can be either pro- or anti-growth in distinct settings, geranylgeranlated RhoB seems to display consistent anti-growth activity [15,45,99]. Mechanistic studies have also indicated that RhoB regulates Cyclin B1 after FTI treatments are initially administered [100]. From this study, the authors illustrated that, in their system, suppression of Cyclin B1 by RhoB was required for FTIs to induce apoptosis.

Unfortunately, the efficacy of FTIs in cell culture and mouse studies has not yet been translated into a positive clinical response and therefore have diminished from the spotlight in anti-cancer therapies over the past several years [101]. Various phase II clinical trials for tipifarnib (R115777) have been conducted in patients with pancreatic cancer, breast cancer, and non-small cell lung carcinoma [102,103,104]. In these trials, no evidence of antitumor activity was observed in pancreatic [102] and non-small cell lung cancer [103], and only about 12% of the patients responded in the advanced breast cancer study [104]. Despite the underwhelming results in the pancreatic, breast, and lung cancers, tipifarnib did go on to phase III studies in colorectal cancer, however, this study reported no significant effects when compared to a placebo [105]. Another FTI, lonafarnib (SCH66336), was in phase II trials for urothelial and colorectal cancers with no advantageous response [106,107]. In a phase I clinical trial for lonafarnib, only one partial response was observed in a patient who was previously treated for metastatic non-small cell lung cancer [108]. There are many potential reasons that might explain why FTIs were more successful in preclinical studies, but lacked any beneficial results against solid tumors. One consideration is that although Ras is important during oncogenic transformation [109], additional mutations must also occur for cancer progression. Because Ras might not be the single driving force in many cancers where FTIs were tested, additional therapies in combination with FTIs might offer a more advantageous outcome, such as taxanes or signal transduction inhibitors of the PI3K-AKT pathway. Pre-clinical data also argue that FTIs may be valuable as an initial chemosensitizer or radiosensitizer [110,111,112], even if they lack efficacy as primary therapeutics. Another huge problem with the mechanism of action for FTIs is that most of their cellular targets for their anti-proliferative effects have not yet been thoroughly established. RhoB was the first established non-Ras target of FTIs for which there was significant evidence that its altered prenylation state was required for the FTI mechanism of action to reverse the Ras-transformed phenotype [113]. Although RhoB has emerged as a key FTI target, its role has remained controversial and must be studied more extensively and further validated in pre-clinical models. Many of these studies were performed years ago and any new signs of evidence involving RhoB in the FTI anti-cancer response has remained unresolved. It is possible that the failure of FTIs in the majority of these clinical trials has contributed to an overall reduced interest in studying the function RhoB. Establishing the precise function of RhoB in the antineoplastic mechanism of FTIs could initiate the development of second generation therapeutics that more efficaciously and directly target the relevant effector pathways. 

## 10. Conclusions and Future Perspectives

These paradoxical observations collectively suggest that the functions of RhoB in cancer are highly context dependent and cell-type specific. Several studies suggest that RhoB plays two distinct and opposing roles in the context of tumor initiation versus tumor progression and aggressiveness [61,72]. In breast tumorigenesis, it was shown that RhoB acts as a tumor suppressor until the tumor vasculature is established, in which then the tumor promoter function of RhoB in endothelial cells override its negative impact in cancer cells [61]. Additionally, in skin cancer, RhoB was shown to favor the early stages of oncogenesis while at the same time limiting tumor aggressiveness [72]. It is obvious that RhoB plays a critical role in many cancers; however, it is crucial that future studies consider RhoB expression or activation in a stage-specific (initiation, tumor growth, and metastasis) manner. We can conclude from the studies mentioned above that RhoB exhibits many unique features that differ from its close relatives: RhoA and RhoC. Due to these differences, we cannot simply predict similar physiological functions based only on the structural similarities. Because of recent studies showing differences in RhoB function during tumor progression, it is crucial that we not only look at how RhoB contributes to tumor growth, but also its role in the ability of cells to leave the primary tumor and invade into other organs in the body. Moreover, to our knowledge, few studies have been published thus far utilizing spontaneous metastasis mouse models to study the role of RhoB in cancer progression. These models of spontaneous metastasis more accurately recapitulate the microenvironmental obstacles that metastatic cells encounter in humans and are therefore a more physiologically relevant system to use. Elucidating the mechanisms that regulate RhoB in the tumor microenvironment are key in understanding its opposing roles in various cancers and can potentially provide the means to develop more targeted, patient-specific therapies in the clinic.

## Figures and Tables

**Table 1:** A summary of the role of RhoB in several cancer types.

Cancer Type	Oncogene	Tumor Suppressor
Bladder		[77] ^‡^
Brain	[52] ^§^, [53], [85], [57] ^§^	[73] ^‡^, [74]
Breast	[58] ^‡^, [59] ^‡^, [60] ^§,^^‡^, [61] *^,§^	[61] *^,§^
Cervical	[68]	[78] ^‡^
Colorectal		[79] ^‡^
Gastric		[80] ^‡^
Head and Neck		[81] ^‡^
Kidney	[66]	[82] ^‡^
Liver	[67] *^,§,‡^	
Lung	[62] ^§,^^‡^, [63] ^§,^^‡^	[31] ^‡^, [37] ^‡^, [49] ^§,‡^, [86] ^§,‡^, [87]
Lymphoma	[64] ^‡^	[88]
Ovarian		[36] ^‡^, [75] ^§,^^‡^, [76] ^§^
Pancreatic		[15] ^§^, [83] ^§,‡^
Prostate	[65]	
Skin	[69], [70] ^§,^^‡^, [72] ^§,^^‡^	[30] ^§^, [48] ^§^, [71], [72] ^§,^^‡^
Thyroid		[84] ^§,‡^

* Endothelial cells; ^§^ includes in vivo study; ^‡^ includes patient samples.
